# Breaking the Gingival Epithelial Barrier: Role of the *Aggregatibacter actinomycetemcomitans* Cytolethal Distending Toxin in Oral Infectious Disease

**DOI:** 10.3390/cells3020476

**Published:** 2014-05-23

**Authors:** Joseph M. DiRienzo

**Affiliations:** Department of Microbiology, School of Dental Medicine, University of Pennsylvania, 240 South 40th Street, Philadelphia, PA 19104, USA; E-Mail: dirienzo@pobox.upenn.edu; Tel.: +1-215-898-8238; Fax: +1-215-898-8385

**Keywords:** *Aggregatibacter actinomycetemcomitans*, cytolethal distending toxin, gingival epithelial cells, gingival epithelium, gingival fibroblasts, periodontal disease, tissue

## Abstract

The Gram-negative bacterium *Aggregatibacter actinomycetemcomitans* is part of the HACEK group that causes infective endocarditis, a constituent of the oral flora that promotes some forms of periodontal disease and a member of the family of species that secrete a cytolethal distending toxin (Cdt). The family of bacteria that express the *cdt* genes participate in diseases that involve the disruption of a mucosal or epithelial layer. *In vitro* studies have shown that human gingival epithelial cells (HGEC) are native targets of the Cdt that typically induces DNA damage that signals growth arrest at the G_2_/M interphase of the cell cycle. The gingival epithelium is an early line of defense in the oral cavity against microbial assault. When damaged, bacteria collectively gain entry into the underlying connective tissue where microbial products can affect processes and pathways in infiltrating inflammatory cells culminating in the destruction of the attachment apparatus of the tooth. One approach has been the use of an *ex vivo* gingival explant model to assess the effects of the Cdt on the morphology and integrity of the tissue. The goal of this review is to provide an overview of these studies and to critically examine the potential contribution of the Cdt to the breakdown of the protective gingival barrier.

## 1. Introduction

*Aggregatibacter actinomycetemcomitans* is a member of the taxonomic family *Pasteurellaceae* that also includes the genera *Actinobacillus/Aggregatibacter* [1], *Haemophilus*, *Mannheimia* and *Pasteurella*. This species along with *Actinobacillus segnis*, *Actinobacillus aphophilus, Haemophilus influenzae*, *H. parainfluenzae, Cardiobacterium hominis, Eikenella corrodens* and *Kingella kingae*, form the HACEK group of bacteria that cause rare cases of infective endocarditis in children [2]. *Aggregatibacter actinomycetemcomitans* has also been strongly implicated in the development of localized aggressive periodontitis (LAP) and possibly contributes to chronic periodontitis (CP), two derivatives of periodontal disease. The disease is initiated by a persistent polymicrobial infection [3,4] and sustained by interactions between the microbial antagonists and host immune system [5]. This bacterium, along with other members of the pathogenic periodontal microflora, produces a variety of products that directly interact with or damage cells and tissues. However, *A. actinomycetemcomitans* is the only indigenous member of the human oral flora identified to date that expresses complex operons for two cytotoxins—a leukotoxin (Lkt) [6] and cytolethal distending toxin (Cdt) [7]. These toxins have significant potential to contribute to the pathogenesis of periodontal diseases [8].

The *A. actinomycetemcomitans* Cdt is a member of a family of related toxins present in a group of Gram-negative bacterial species that are facultative or microaerophilic and key pathogens in diseases that involve the perturbation of a mucosal (enteritis, gastric ulcers, chancroid) or epithelial (periodontal diseases) layer. By convention the various Cdts are identified by an abbreviated genus and species prefix such as *Aa*Cdt [9]. Studies in my laboratory have been focused on the *Aa*Cdt and the possible role of this toxin in the breakdown of the human gingival epithelium. Perturbation of this tissue is thought to be an early event in periodontal disease. Bacterial sampling of global and country-specific patient populations has shown that a significant proportion of oral isolates of *A. actinomycetemcomitans* carry the *cdt* genetic locus. Strains that have *cdt* gene sequences and exhibit associated cytotoxic activity have been recovered with reasonable frequency from subjects diagnosed with periodontal disease [10,11,12,13,14,15,16]. Systemic Cdt antibodies have been found in periodontitis patients indicating infection with Cdt^+^ strains [17,18,19]. In our studies, all fresh clinical isolates of *A. actinomycetemcomitans* obtained from a large geographically homogeneous population of LAP families contain a chromosomal locus for the Cdt [20,21]. Although some of these isolates have *cdt* gene deletions of various lengths, all members of one restriction fragment length polymorphism (RFLP) cluster group contain a complete *cdt* operon [7]. There was a high statistical correlation between this RFLP group II and conversion of young children from a healthy to diseased periodontal status [22]. More recently, a study of 249 isolates of *A. actinomycetemcomitans* from 200 Ghanian adolescents were screened for serotype, the presence of *cdt* gene sequences and the ability to induce cell cycle arrest of HL-60 cells [23]. Complete *cdt* gene sequences were found in 79% of the isolates examined and all of these isolates exhibited Cdt activity. Fifty-three percent of the Cdt^+^ isolates correlated with attachment loss indicative of LAP. In another recent study, *A. actinomycetemcomitans* isolated from 255 subgingival samples from aggressive and chronic periodontitis and clinically healthy sites in 30 Chinese subjects were screened for only the *cdtB* gene sequence [24]. The *cdtB* gene was detected in isolates from 78% of the aggressive sites, 74% of the chronic sites and none of the healthy sites. Although that study concluded that Cdt^+^ strains may correlate with disease, no attempt was made to confirm that the *cdtB*^+^ isolates produced active toxin. In total these studies support an indirect and less than 100% association between the Cdt and LAP. Thus, an elusive goal has been to link Cdt activity with the manifestation of specific *in vivo* events characteristic of the disease. As best stated in a recent review, “One of the true challenges in the CDT field is to understand the *in vivo* consequences of CDT action during infection” [25]. The goal of this review is to present and critically analyze current information supporting the hypothesis that the *Aa*Cdt plays a role in early events associated with periodontal disease.

## 2. *Aggregatibacter actinomycetemcomitans* Cdt Structure and Function

### 2.1. Cell Surface Recognition

The *cdt* operon resides on the *A. actinomycetemcomitans* chromosome [7]. The three structural genes, *cdtA*, *cdtB* and *cdtC*, are expressed from a polycistronic operon [26,27,28]. The sizes of the *Aa*Cdt gene products are approximately 18–25 kDa (CdtA), 31 kDa (CdtB) and 21 kDa (CdtC). All three proteins are required to produce a toxin that can intoxicate cells and inhibit their proliferation.

**Figure 1:**
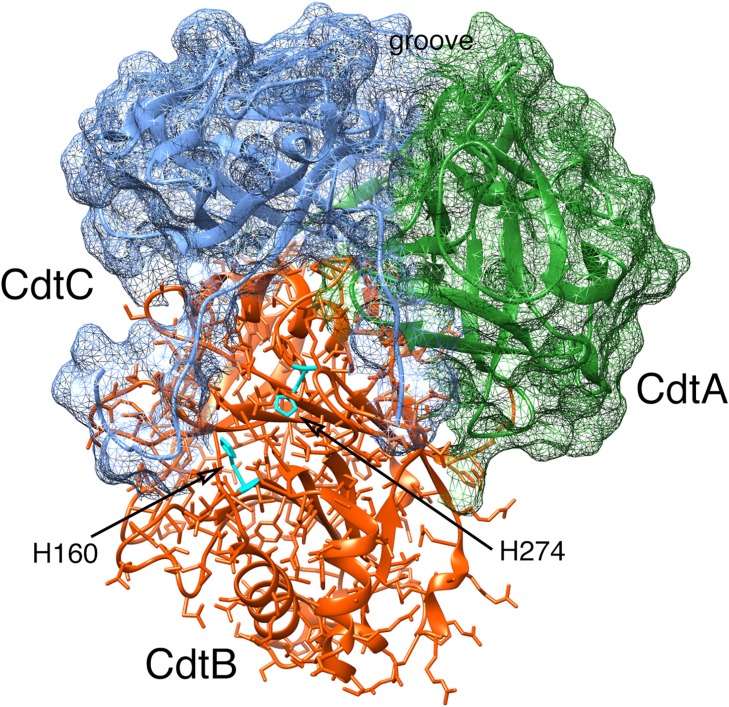
Computer model of the *Aa*Cdt showing the structural relationship of the three subunits: CdtA, CdtB and CdtC. The subunits are depicted as ribbon backbone models using the Protein Data Bank (PDB) file 2F2F, deposited by Yamada *et al.* [29], and UCSF Chimera 1.8.1. Side chains are shown only in CdtB. The surfaces of CdtA and CdtC are shown as mesh models. Two residues, H160 and H274, in the active site of CdtB are required for toxin activity. Abbreviations: Cdt, cytolethal distending toxin.

Based on the crystal structure of the *Aa*Cdt [29], the products of the *cdtA* and *cdtC* genes are predicted to form a heterodimer partially separated by a deep groove which functions as a binding site for a receptor on the target cell surface (Figure 1). Studies using the *Ec*Cdt indicated that CdtA and CdtC recognize N-linked fucose moieties on the surface of HeLa cells [30]. This binding specificity was exploited to employ an ELISA assay, based on the fucose-containing glycoprotein thyroglobulin, to study binding kinetics of the *Aa*CdtA and *Aa*CdtC subunits [29]. Recombinant CdtA alone can bind to cells in culture, based on the results of immunofluorescence experiments [27,30,31] and in an enzyme-linked immunosorbent assay on live cells (CELISA) [32,33]. CdtC has limited amino acid sequence similarity with CdtA [32,33] suggesting, but not proving, a related function. Some studies have shown that recombinant CdtC binds to HeLa and HEp-2 cells in culture and in the CELISA [30,33,34]. In contrast, other studies have found that CdtC binds poorly to CHO cells [32]. In addition to these data which showed strong binding of the Cdt to membrane glycoproteins, a separate study found that recombinant *Aa*Cdt binds to gangliosides GM1 [Galβ(1,3)GalNAcβ(1,4)[NeuAcα(2,3)]-Galβ(1,4)Glc-ceramide], GM2 [GalNAcβ(1,4)[NeuAcα(2,3)]-Galβ(1,4)Glc-ceramide] and GM3 [NeuAcα(2,3)Galβ(1,4)Glc-ceramide] [35]. GM3 is a component of membrane lipid rafts [36]. Lipid rafts are areas of the cell membrane that are enriched for cholesterol and have been implicated in Cdt binding primarily via a cholesterol recognition/interaction amino acid consensus (CRAC) motif in CdtC [37,38]. Application of a novel haploid genetic screening method, using a derivative of a chronic myeloid leukemia cell line, found that recombinant *Ec*Cdt binds to the G-protein-coupled receptor TMEM181 [39]. In spite of the information obtained from these binding kinetic studies, the identity of a specific cell surface receptor for the *Aa*Cdt remains elusive. It is becoming apparent that the species-specific Cdts, or subgroups containing the *Aggregatibacter/Haemophilus* and *Escherichia/Salmonella/Campylobacter/Helicobacter* Cdts, may have distinct host cell receptors and mechanisms of intoxication [38]. However, the reason why the various species-specific Cdts have different properties has not yet been deciphered at the molecular level. 

### 2.2. Cytotoxicity

**Figure 2:**
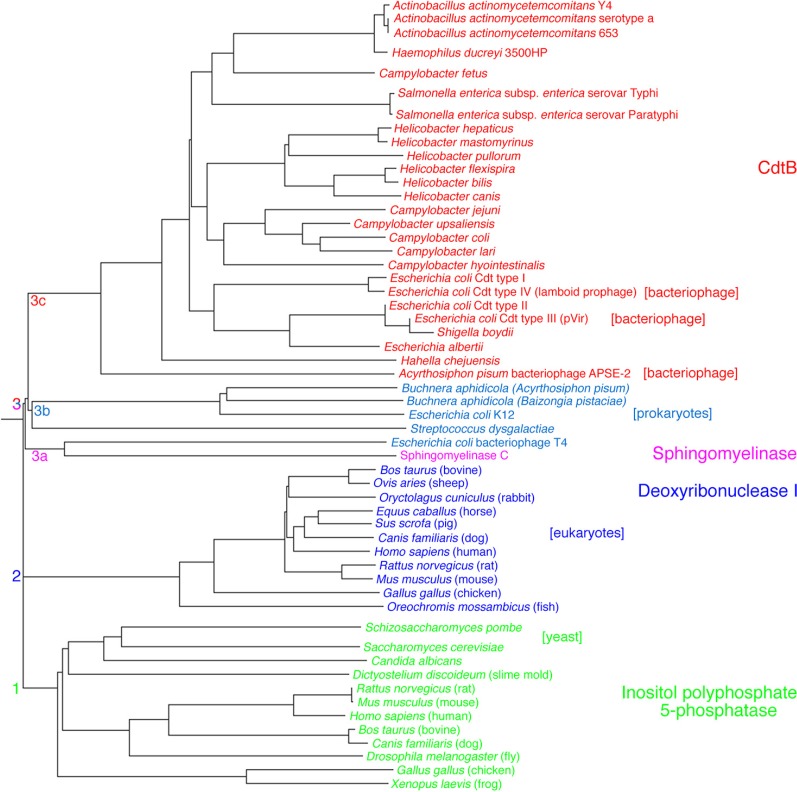
Rooted phylogenetic tree of the deduced amino acid sequences of a representative group of deoxyribonucleases (DNase I), inositol polyphosphate 5-phosphatases and sphingomyelinase. The tree was constructed with PHYLIP 3.6. The lengths of the lines show relative genetic distances.

The third subunit, or product of the *cdtB* gene, represents the biologically active component and has to enter cells to elicit virulent effects. A broad comparison of deduced amino acid sequences shows that CdtB belongs to a superfamily of enzymes that includes the endonucleases, exonucleases, sphingomyelinases, and inositol polyphosphate 5-phosphatases [40]. The phylogenetic relationships imply that CdtB appears to be most closely related to prokaryotic and bacteriophage deoxyribonuclease I and sphingomyelinase C (Figure 2; subclusters 3a, 3b and 3c) with broader similarities to phosphatidylinositol-3,4,5-triphosphate phosphatase and eukaryotic deoxyribonuclease I (clusters 1 and 2). Based on these phylogenetic relationships CdtB has the potential to exhibit multiple enzymatic activities *in vitro* and *in vivo*. 

#### 2.2.1. Primary Activities Associated with CdtB

DNA damage in the form of double-strand breaks, as assessed by pulsed-field gel electrophoresis, is one of the CdtB-related activities observed in sensitive cell types exposed to the heterotrimer [31,41,42]. CdtB requires divalent cations for DNA-nicking activity [43,44] with an optimum concentration of 50 mM MgCl_2_, CaCl_2_ or MnCl_2_ for the *Aa*CdtB [45]. Mutating conserved deoxyribonuclease active site residues His160 and His274 in the *Aa*Cdt [46], or their equivalent His residues in the other species-specific CdtBs, result in the loss of both *in vitro* nuclease activity and cell cycle arrest [44,47]. Therefore, CdtB can act as a neutral, cation-dependent deoxyribonuclease I-like nuclease in most susceptible cell types examined [31,45]. This DNA-damaging activity is unusual for a bacterial cytotoxin and classifies the Cdt as a genotoxin [48].

The fact that CdtB is a member of the large metaloenzyme superfamily led to the proposal that the toxin acted upon susceptible cells or cell lines by dephosphorylation of the Wee1 kinase or Cdc25 phosphatase [49]. These two enzymes are important for regulating the phosphorylation of tyrosine residues in Cdc2 kinase. An increase in Cdc2 phosphorylation leads to cell cycle arrest. This rationale was used to form the hypothesis that the primary mode of action of CdtB is that of a phosphatase rather than that of a nuclease. Additional support for the phosphatase mode of action came from studies showing that recombinant *Aa*CdtB behaved as a phosphatidylinositol-3,4,5-triphosphate phosphatase in lymphoid cell lines [50]. It was proposed that the action of the Cdt in lymphocytes causes a depletion of phosphatidylinositol-3,4,5-triphosphate which in turn inactivates the Akt pathway leading to cell cycle arrest and apoptosis. Human macrophages exposed to the *Aa*Cdt experience inhibition of phosphatidylinositol-4,5-bisphosphate 3-kinase signaling pathway and an associated increase in glycogen synthase kinase 3β affecting production and secretion of the cytokines interleukin (IL)-1β), IL-6 and tumor necrosis factor (TNF)-α [51]. However, the macrophages did not exhibit Cdt-induced apoptosis.

#### 2.2.2. Cell Signaling Activities

It has been reported that the *Aa*Cdt stimulates the synthesis of IL-1β, IL-6, and IL-8 in human peripheral blood mononuclear cells (PBMC) [26] and IL-8 in *Cj*Cdt-treated human embryo intestinal epithelial cells (INT407) [52]. Nitric oxide production was altered in macrophage cultures due to the presence of the *Aa*Cdt [53]. Exposure of macrophages to the toxin also resulted in increased levels of IL-1β, IL-10, IL-20 and TNF-α. These findings indicated that the *Aa*Cdt may modulate macrophage function *in vivo* by upsetting the equilibrium between pro-inflammatory and anti-inflammatory cytokines. However, recombinant *Aa*Cdt failed to stimulate IL-1β in human periodontal ligament fibroblasts but up-regulated IL-6 and the receptor activator of NF-κB ligand (RANKL) in these cells [54,55]. Another report showed that HGF challenged with whole *A. actinomycetemcomitans* bacteria exhibited increased expression of IL-6 and IL-8 [56]. This increase in expression was not attributed to a specific product but the Cdt is a reasonable candidate. RANKL was also up-regulated in response to the *Hd*Cdt in a T-lymphocyte leukemia cell line (Jurkat) [57]. RANKL is a member of the TNF ligand superfamily and binds to its associated receptor RANK on osteoclast progenitor cells [58]. This action leads to differentiation of the progenitor cells into bone-resorbing osteoclasts [59]. Since expression of these molecules is involved in the induction of osteoclast differentiation they have a central role in the regulation of bone resorption that is a key feature of periodontitis [60,61].

#### 2.2.3. Cell Cycle Arrest as a Result of DNA Damage

**Figure 3:**
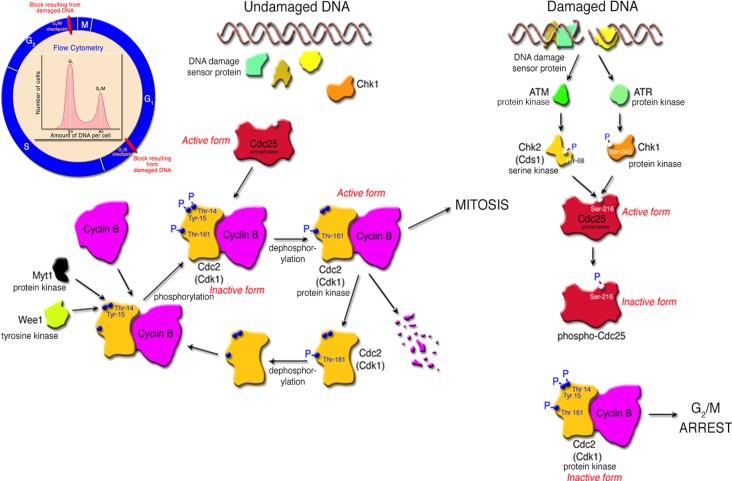
Checkpoint pathway, in response to DNA damage, that leads to growth arrest at the G_2_/M interphase of the cell cycle in epithelioid cells. The inset illustrates the standard DNA damage checkpoints at G_1_/S and G_2_/M in the cell cycle. Cell cycle arrest at G_2_/M can be measured by flow cytometry or cell sorting. Populations that are arrested at G_2_/M accumulate cells that have a 4*n* DNA content.

Early studies of the Cdt showed that the toxin was active against HeLa, Hep-2, CHO and Vero cells [26,62,63,64,65,66]. Caco-2, a well-known human colon carcinoma cell line [67], and two human keratinocyte cell lines, HaCat [68,69] and Henle-407 (intestinal epithelial cells) [47], experience cell cycle arrest when exposed to the Cdt. DNA damage induced by CdtB typically leads to cell cycle arrest due to activation of checkpoint responses. Cell cycle checkpoints guard the faithful progression to cell division when DNA damage is detected. Subsequent growth arrest results from the block in cell cycle progression that occurs in response to the Cdt at the either the G_1_/S or G_2_/M phase transitions. The pathways for cell cycle arrest in response to the Cdt appear to be cell-type specific and have been outlined in various reviews [70,71,72]. Epithelial-like cell lines such as HeLa [66,67,73], HEp-2 [26] and Caco-2 [67] as well as immortalized human gingival keratinocytes [74] are arrested at the G_2_/M phase transition (Figure 3). The key inhibitory step in this Cdt-altered pathway is the failure of protein phosphatase Cdc25 to dephosphorylate checkpoint protein kinase Cdc2 (Cdk1). Toxins that modulate the eukaryotic cell cycle have been termed cyclomodulins [72,75].

HEp-2 and KB cell lines, generally thought to have originated from epidermoid carcinomas of the mouth and larynx, respectively, are sensitive to the *Aa*CDT [76]. This is particularly significant since the KB cell line had been used extensively in *A. actinomycetemcomitans* invasion studies [77,78,79]. Furthermore, an immortalized human orolabial cell line, GMSM-K [simian virus (SV40) transformed] [80], is particularly sensitive to the Cdt and served as an early epithelial cell model for *Aa*Cdt studies prior to our ability to culture primary human gingival epithelial cells (HGEC) [31,81]. Interestingly, this cell line was arrested at the S phase of the cell cycle in response to the toxin.

Primary cells isolated from the epithelial layers of human gingival tissue, obtained from routine crown-lengthening procedures, had an epithelioid morphology, bound a monoclonal antibody specific for epithelial cell adhesion molecule (Ep-CAM) and failed to bind an anti-fibroblast CD90/Thy-1 antigen (Ab-1) antibody [82]. These cells arrested at the G_2_/M interphase of the cell cycle when exposed to recombinant *Aa*Cdt for 18 h. There was no effect on the cell cycle when these cells were exposed to toxin containing the mutated CdtB subunit (CdtAB^H160A^C) for up to 36 h. 

In our hands, oral cells of mesenchymal origin such as human periodontal ligament fibroblasts (HPLF) [31], human gingival fibroblasts (HGF) [82] cementoblasts [81] and osteoblasts (unpublished observations) appear to be resistant to the DNA-damaging and cell cycle arresting effects of the Cdt. Cells cultured from the connective tissue layer of gingival tissue exhibited morphology typical of fibroblasts and bound the CD90/Thy-1 (Ab-1) antibody. Cultures of these HGF failed to accumulate cells having a 4*n* DNA content upon continuous exposure to recombinant *Aa*Cdt for up to 96 h [82]. Others have reported a non-lethal inhibition of proliferation of HPLF attributed to the *Aa*Cdt [83] and, in a subsequent study, cell cycle arrest at both G_1_ and G_2_/M in HPLF and HGF [84]. It is unclear why there are conflicting results. However, the reproducible ability of the *Aa*Cdt to inhibit the proliferation of primary HGEC suggests that a predominant activity of the toxin *in vivo* is in damaging the protective epithelial barrier that is important in preventing the initial development of periodontal disease.

## 3. Breakdown of the Gingival Epithelial Barrier

### 3.1. Gingival Tissue

Since it was observed that epithelial cells are highly susceptible to Cdt-mediated intoxication they are likely to be natural targets of the *Aa*Cdt in the noninflamed or healthy oral cavity. Gingival epithelium is made up of three components: oral (OE), sulcular (SE) and junctional epithelium (JE) [85]. The OE is a keratinizing form of epithelium and provides a very effective physical barrier to microbial invasion of the underlying gingival connective tissue (CT). In contrast, junctional epithelium, and in some instances sulcular epithelium, are non-keratinizing forms of epithelium. Therefore, the junctional and sulcular epithelium are semi-permeable allowing the transport of soluble macromolecules from the gingival sulcus into the underlying connective tissue. The basal cell layers of all three types of gingival epithelia are composed of rapidly proliferating cells that migrate towards the outer surface of the tissue. In this process the cells leave the cell cycle and start to differentiate. Relative to oral epithelium and sulcular epithelium this process is associated with loss of expression of the cytokeratins K5, K14 and K19 found in basal cells. Suprabasal junctional epithelial cells continue to express K19 [85]. Thus, junctional epithelium is considered to be a less well-differentiated type of epithelium. Subsequently, the suprabasal cells of all three layers start to express different patterns of alternative cytokeratins. Terminally differentiated cells found in keratinizing epithelia also express a unique group of proteins that includes filaggrin, involucrin and loricrin [86,87].

The gingival epithelial surface is under constant assault by biofilm-forming bacteria, including *A. actinomycetemcomitans*, which colonize the human oral cavity. Colonization of the subgingival microenvironment places *A. actinomycetemcomitans* in close proximity to junctional and sulcular epithelium cells. This tissue layer creates an early line of defense against microorganisms by forming a barrier that blocks or, at least minimizes, bacterial invasion of the underlying connective tissue [88,89]. Bacterial products, along with factors released from recruited inflammatory cells, induce degradation of connective tissue and bone resulting in damage to the supporting structures of the teeth. Therefore, breakdown of this epithelial barrier early in the development of periodontal disease has significant consequences for oral health. Two key observations provided the rationale to test the effects of the *Aa*Cdt in a human *ex vivo* model. (i) All members of the Cdt family are produced by bacterial species associated with diseases that involve tissue lined with epithelial cells. Additionally, (ii) epithelioid cells are particularly sensitive to the anti-proliferative related activities of the toxin.

### 3.2. Damaging Effects of Cdt on Human Gingival Tissue

Previous studies had shown that human gingival explants (HGX) could survive in the laboratory with the basal epithelial cells exhibiting intense proliferative activity for up to 48 h [90]. No evidence of degenerative changes or de-differentiation of cells in the explants was detected for up to four days of incubation [91]. We found that clinically healthy tissue originating from routine crown lengthening surgeries conducted on physically healthy adults maintained their morphological integrity in tissue culture medium for up to 36 h *ex vivo* [82]. Histological staining of untreated tissue sections with hematoxylin and eosin (H&E) showed that the oral epithelium, rete pegs (RP) and connective tissue appeared normal for a minimum of 24 h. However, in some sections a slight mechanical separation of the keratinized surface layer (KL) was observed after 18 h of incubation.

#### 3.2.1. Changes in Tissue Morphology and Cellular Organization

The epithelial cell layers of gingival explants exposed to recombinant *Aa*Cdt for 18 h exhibited pronounced separation of the keratinized surface layer, extensive disruption of the epithelial layers marked by a swollen appearance or thickening and a loss of structural integrity of the rete pegs [82]. Cells in the oral epithelium were dramatically distended and interstitial spacing between the cells increased dramatically indicating an apparent loss of cell junctions. The morphological changes were reminiscent of the histology of gingival tissue obtained from patients who had clinical signs of periodontal disease. Very similar effects were observed when recombinant *Aa*Cdt was topically applied to the palatal gingival sulcus of the maxillary molars of rats [92].

To confirm that the Cdt-induced changes in morphology were specific to the toxin, gingival explants were also treated with Cdt reconstituted with the mutated CdtB subunit (CdtAB^H160A^C). Gingival explants exposed to CdtAB^H160A^C for up to 36 h appeared to be unaffected. Both the wild-type and mutated CdtB subunit were found predominantly in the oral epithelium as detected by immunofluorescence. A minimal amount of CdtB appeared to reach the connective tissue that most likely was due to the epithelial damage induced by the toxin. 

#### 3.2.2. Disruption of Cell Junctions

Abnormal physiological processes associated with disease can disrupt cell-cell contact leading to degradation of tissue organization and structure [93]. Perturbations in the regulation of cell-cell junction stability and dynamics impact the barrier properties of epithelial layers and can have detrimental effects on tissue remodeling [94].

Changes in tissue morphology were observed following exposure to the *Aa*Cdt that upon general appearance could be predicted to be due to a loss of cell-cell contact. Contact between epithelial cells, essential for tissue integrity, is maintained by complexes consisting of transmembrane, cytosolic and cytoskeletal proteins. The two major types of junctions relevant to epithelial cells located in oral epithelium are zonula occludens or tight junctions which contain the transmembrane proteins claudin and occludin and the cytoplasmic protein ZO-1 [95] and zonula adherens or adherens junctions which are complexes that interact with intracellular scaffolding and signaling molecules [95,96,97,98]. Two other types of cell junctions include macula adherens or desmosomes which are transmembrane glycoproteins (desmogleins) found in stratified squamous epithelia such as that found in the soft tissue lining of the mouth, surface layer of the skin, the esophagus and vagina [93] and gap junctions which are made up of paracrystalline connexin channels in a multitude of tissues [93,99]. 

The presence of tight junctions in human gingival epithelium was suggested previously but not unequivocally demonstrated [100,101]. Microscopic evidence of tight junctions in human gingival biopsies was reported [102]. However, there were areas in the tissue where tight junctions were not detected. In another study using cultured human gingival keratinocytes only a minimal increase in transmembrane electrical resistance (TER) was detected as the cells formed multilayers and this increase was not altered by exposure of the cells to either *Porphyromonas gingivalis* or *A. actinomycetemcomitans* for up to 24 h [103]. It has been reported that *P. gingivalis* is capable of degrading epithelial cell-cell junction complexes [104]. Attempts were made to use changes in TER to measure the effect of the Cdt on tight junction maintenance in post-confluent cultures of HGEC isolated from the HGX [105]. However, this approach was unsuccessful because the HGEC apparently failed to form tight junctions *in vitro*. This was confirmed by the low concentration of claudin 1, a primary component of tight junction filaments [106], in the cells as determined by immunofluorescence. Attempts to identify tight junctions in the gingival explants were also unsuccessful since technical limitations preclude the measurement of TER in tissue and claudin 1 was not readily detected.

Ye *et al.* [107] reported a relatively high level of expression of E-cadherin in the basal cell layer of healthy human gingiva. Therefore, E-cadherin was used as a marker to examine the effects of the recombinant *Aa*Cdt on adherens junctions *in situ* [105]. E-cadherin localized predominantly at the plasma membrane, outlining epithelial cell-cell contact zones in the oral epithelium of untreated HGX. E-cadherin staining was absent on the surface of those epithelial cells that lined the leading edge of the rete pegs that were in contact with the basement membrane. The absence of E-cadherin at this site was most likely due to the fact that these cells attach to the basement membrane via hemidesmosomes. Staining with wheat germ agglutinin confirmed the presence of intact epithelial cell membranes in those cells that were adjacent to the basement membrane. These results supported previous observations that normal oral epithelium showed strong pericellular staining of E-cadherin in the basal and suprabasal cell layers but not in the basal aspect of keratinocytes adjacent to the basement membrane [108]. 

Exposure of the HGX to the *Aa*Cdt resulted in a statistically significant increase in the E-cadherin mRNA expression level in 50% of HGX and 66% of HGEC cultures obtained from 19 clinically healthy subjects. A pronounced increase in the relative fluorescence of E-cadherin in the oral epithelium of the tissue correlated with an increase in E-cadherin mRNA expression in those samples examined. The protein was detected throughout the cytosol indicating extensive intracellular redistribution. E-cadherin was now detected on the surface of those cells that lined the rete peg-basement membrane border in the Cdt-treated HGX. Not only did this observation confirm that Cdt induces redistribution of E-cadherin within the epithelial cells but also indicates that the toxin causes detachment of basal cells from the basement membrane. Exposure of HGX to CdtAB^H160A^C minimally affected the intensity of E-cadherin staining but not to the extent of that observed in HGX incubated with wild type toxin. HGX from patients that had clinical signs of periodontal disease exhibited relatively high levels of E-cadherin in the spinous layer.

#### 3.2.3. Changes in Expression of Intracellular Scaffolding Proteins

Adherens junction structure relies on dynamic interactions among E-cadherin, catenin complexes and the actin cytoskeleton [94,97,109,110,111,112]. The accumulation of F-actin assemblies having a morphology similar to that of actin stress fibers were observed in CHO cells treated with recombinant *Ec*Cdt [113]. This change in the structural apparatus of the cells correlated with an inhibition of cell division and, therefore, further demonstrated the influence of the Cdt on the regulation or function of cell cycle-dependent events leading to cytokinesis.

We found that 25% of HGX and 50% of 19 HGEC cultures examined showed a statistically significant increase in β-catenin mRNA expression after exposure to the recombinant *Aa*Cdt [105]. Actin mRNA expression increased in 63% of HGX and 83% of the HGEC cultures following Cdt-treatment. The β-catenin concentration in the spinous layer of selected HGX was elevated, relative to that in untreated tissue, following exposure to the recombinant Cdt for 18 h. There was also a marked increase in actin staining as early as 6 h after the addition of the toxin. HGX from patients that had clinical signs of periodontal disease exhibited relatively high levels of β-catenin in the spinous layer.

The Cdt induces significant changes in both the distribution and expression of the component proteins of the adherens junction complex in both HGEC and HGX from a majority of samples examined. These effects may be associated with the cellular distention caused by the toxin since stretching of the cells would be expected to exert physical effects on the membrane and cell surface. The variability detected among the tissue samples in response to the Cdt could be due to a multitude of environmental and/or donor-specific factors. The precise mechanism by which the Cdt affects the expression and redistribution of the adherens junction complex is not known at this time.

### 3.3. Gingival Breakdown Model

**Figure 4:**
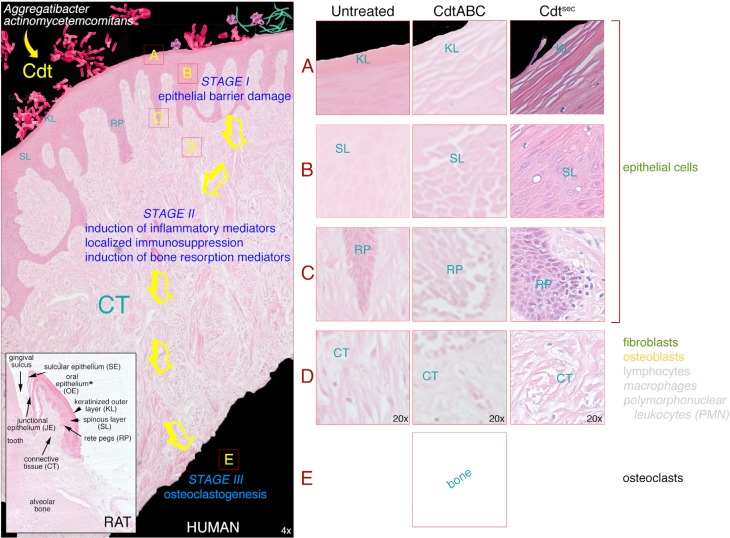
Human gingival explant (HGX) model of the effects of secreted *Aa*Cdt on tissue obtained from clinically healthy subjects. HGX were untreated or exposed to recombinant wild-type *Aa*Cdt (CdtABC) or native toxin secreted by the bacterium (Cdt^sec^). Tissue sections were stained with hematoxylin and eosin. Abbreviations: KL, keratinized layer; SL, spinous layer; RP, rete peg; CT, connective tissue. Inset shows the overall histology of the gingival tissues and various types of epithelium (junctional, sulcular and oral) in the upper jaw of a rat. The tissue was decalcified, sectioned and stained with hematoxylin and eosin.

Our studies provide compelling evidence for the contribution of the *Aa*Cdt to the breakdown of the human gingival epithelium. As noted in a recent review, “These results, showing a direct damage of human oral epithelium and mucosa by CDT, indicate a clear role for AaCDT in the pathogenesis of periodontitis.” [114]. Based on the *ex vivo* effects of the toxin on HGX a model is proposed detailing the role of the Cdt in breakdown of the gingival epithelial barrier and subsequent contribution to events leading to the development of periodontal disease (Figure 4). 

There is experimental evidence indicating that the Cdt is secreted as an active heterotrimer by *A. actinomycetemcomitans* [115]. This is indirectly supported by a myriad of studies that have used cell-free medium from exponential or stationary phase cultures of *A. actinomycetemcomitans* as a source of toxin for cell culture experiments and activity assays. Therefore, *A. actinomycetemcomitans* can colonize the oral cavity and continuously release the Cdt. It is assumed that the *cdt* genes are constitutively expressed because there is no direct evidence to show either transcriptional or post-transcriptional regulation. It is possible that the splicing of intervening sequences from *cdt* transcripts may be a mechanism of regulating the expression of the *Aa*Cdt proteins [116]. However, there is no proof of such a mechanism of post-transcriptional control. It has been proposed, based on the analysis of mutations in the *luxS* gene, that the *Cj*Cdt is under the regulation of autoinducer AI-2 (furanosyl borate diester) [117]. However, there is no further evidence to support this mechanism in the regulation of the *Aa*Cdt. 

Since the Cdt is a secreted product the bacterium does not have to be in direct contact with the gingival epithelium or epithelial cells in the tissue to impart damage. The secreted toxin can compromise the barrier function of the gingival epithelium by binding to and intoxicating the keratinized epithelial cells at the surface of the tissue, thereby disrupting the keratinized surface layer (Stage IA). The specific molecular mechanism of damage to the keratinized cells in this layer is not known at this time because these are non-proliferating cells. As the cells in the spinous layer start to differentiate their rate of proliferation slows and they eventually exit the cell cycle as they go through terminal differentiation becoming part of the keratinized layer. The keratinized layer appears to slough off the HGX in sections or thin strips in response to the toxin. Disruption of the keratinized layer creates an environment in which the Cdt has access to the highly proliferating cells in the oral epithelium. Cells within the deeper layers of epithelium should therefore be good targets for the toxin since they continuously proliferate prior to terminal differentiation as they migrate towards the epithelial surface [118]. 

**Figure 5:**
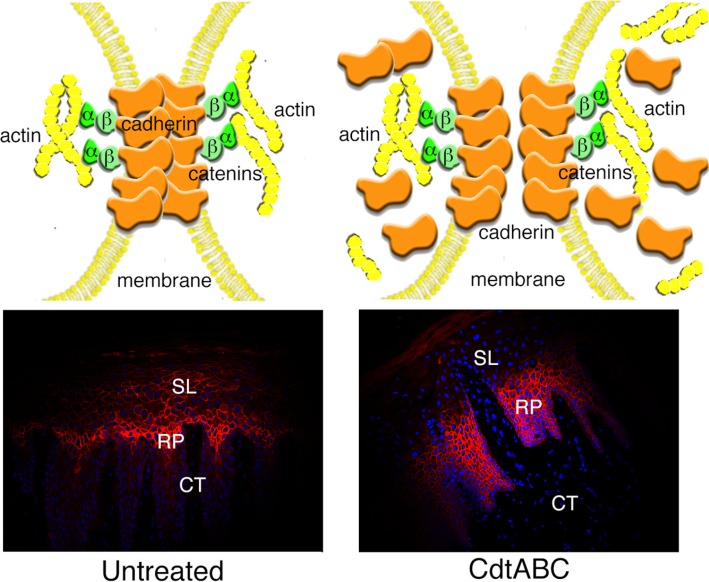
Model of the effects of the *Aa*Cdt on gingival epithelial cell adherens junctions *in situ*. E-cadherin was detected with a polyclonal antibody conjugated to fluorescein (red fluorescence) in untreated HGX and explants exposed to recombinant *Aa*Cdt. Cell nuclei were stained with DAPI (blue fluorescence). Abbreviations: SL, spinous layer; RP, rete peg; CT, connective tissue.

Based on the analysis of the effects of the Cdt on HGEC isolated from the same gingival tissue as that used in the *ex vivo* experiments, the *Aa*Cdt most likely induces irreversible cell cycle arrest (G_2_/M) of the epithelial cells, *in situ*, in the spinous layer and rete pegs (Stage IB and IC). This is evidenced by pronounced swelling of the tissue and enlargement or distention of the epithelial cells. Cell-cell contact in the tissue appears to be significantly disrupted by an increase in the interstitial spaces between cells most likely due to the redistribution of adherens junction and scaffolding proteins (Figure 5). The Cdt-induced changes related to cell proliferation within the spinous layer and rete pegs may be secondary to effects on cell-cell junctions. Distention of the HGEC and dissolution of cell junctions could promote more wide-spread penetration of the toxin throughout the tissue. Breakdown of the oral epithelium would also facilitate entry of other members of the periodontal microflora and a host of bacterial products into the connective tissue.

Based on the assessment of the affects of the *Aa*Cdt on cultured HPLF and HGF in our studies [31,82], the toxin may not significantly affect the proliferation of the resident fibroblasts and osteoblasts in the extracellular matrix. Our results differed from those of Belibasakis *et al.* [83,84]. However, the toxin may induce the fibroblasts to release pro-inflammatory cytokines such as IL-6 and mediators of bone resorption such as RANKL in Stage II as shown by that group in *in vitro* studies [57].

Epithelial cells also function as part of a signaling network that alerts inflammatory cells to a microbial assault [119,120]. In response to the epithelial cell damage and cell signaling induced by the Cdt, inflammatory cells such as lymphocytes, macrophages and polymorphonuclear leukocytes (PMN) can infiltrate and increase in number in the connective tissue. The Cdt stimulates these cells to increase cytokine production and may lead to localized immune suppression by inhibiting the proliferation of the infiltrating T-lymphocytes and macrophages exacerbating the immune response. Human CD4^+^ and CD8^+^ T lymphocytes as well as monocytes undergo cell cycle arrest, without cell distention in the G_2_ phase when treated with extracts containing the *Aa*Cdt [121,122]. Lymphocytes treated with recombinant *Aa*CDT undergo apoptosis via activation of the caspase cascade [123]. Furthermore, there is evidence for a role for the apoptosis regulator Bcl-2 [124] in the apoptotic process. These results were confirmed by Nalbant *et al.* [121] who also found that Cdt-induced apoptosis involves the up-regulation of Fas and FasL, down-regulation of Bcl-2, activation of caspase-3 and was dependent on the presence of monocytes. Binding of the Fas ligand (FasL) to the Fas receptor (CD95) on target cells is one of the pathways by which apoptosis is initiated [125]. Use of a human leukemic cell line (MOLT-4) indicated that two independent pathways, caspase-dependent (early) apoptotic cell death and caspase-independent (late) cell death, may be involved in Cdt-induced death in some types of T-cells [126,127]. Inhibition of immune cell proliferation by cell cycle arrest and/or apoptosis may be a contributing factor to immune suppression that would allow the continued colonization of infected sites by *A. actinomycetemcomitans* or other periodontal pathogens in periodontal disease. In addition, results of *in vitro* experiments with U937 cells, a macrophage-like cell line, suggested that macrophages are potential *in vivo* targets of the toxin [128]. The *Aa*Cdt may disrupt macrophages by inhibiting phagocytic activity, modulating nitric oxide production and altering the expression of pro-inflammatory and anti-inflammatory cytokines [53,129]. However, it should also be noted that *A. actinomycetemcomitans* resides in a biofilm external to the tissue and may be protected to some degree from immune surveillance and clearance by phagocytosis.

Finally, the Cdt-induced mediators, released in Stage II, that stimulate differentiation of progenitor cells into osteobclasts and endotoxin or lipopolysaccharide (LPS) produced by Gram-negative bacteria can lead to osteoclastogenesis (Stage III) which is the defining event in periodontal disease.

## 4. Concluding Statements

Attempting to provide unequivocal proof that a particular microbial product makes a major contribution to human infectious disease is a daunting undertaking. Periodontal diseases pose a particularly challenging problem due to the complex polymicrobial etiology and the uniqueness of this disease to the human condition. The use of gene knock-out mutants in animals has been employed to study some oral microbial virulence factors. However, existing animal models do not seem to be sufficient to address the unique genotoxic effects of the Cdt. Although the HGX model for characterizing the possible role(s) of the *Aa*Cdt in disease-associated tissue damage is not a perfect one, it provides a novel experimental approach to begin to address these important questions. In addition, there are unique advantages in being able to correlate data from an examination of the effects of the toxin on cells *in situ* with data from experiments that characterize the inhibitory pathways affected by the toxin in the same cells in culture. 

The characteristics of the Cdt make a compelling case that this bacterial product is a relevant virulence factor in *A. actinomycetemcomitans-*associated diseases. Clearly, the *Aa*Cdt has the potential to affect cells important to the protection and integrity of the periodontium. In conjunction with the ability of some strains of *A. actinomycetemcomitans* to invade epithelial cells, the Cdt could greatly enhance the ability of this bacterium, as well as others, to bypass the physical barrier established by the gingival epithelium. Damage to a protective epithelial layer makes the Cdt a key participant in the earliest stages of the development of some forms of periodontal disease and serves as a paradigm for the other members of the Cdt family.
